# A Periplasmic Lanthanide Mediator, Lanmodulin, in *Methylobacterium aquaticum* Strain 22A

**DOI:** 10.3389/fmicb.2022.921636

**Published:** 2022-06-23

**Authors:** Yoshiko Fujitani, Takeshi Shibata, Akio Tani

**Affiliations:** ^1^Institute of Plant Science and Resources, Okayama University, Okayama, Japan; ^2^K.K. AB SCIEX, Tokyo, Japan

**Keywords:** lanmodulin, lanthanide, methanol dehydrogenase, *Methylobacterium* species, membrane vesicles

## Abstract

*Methylobacterium* and *Methylorubrum* species oxidize methanol *via* pyrroloquinoline quinone-methanol dehydrogenases (MDHs). MDHs can be classified into two major groups, Ca^2+^-dependent MDH (MxaF) and lanthanide (Ln^3+^)-dependent MDH (XoxF), whose expression is regulated by the availability of Ln^3+^. A set of a siderophore, TonB-dependent receptor, and an ABC transporter that resembles the machinery for iron uptake is involved in the solubilization and transport of Ln^3+^. The transport of Ln^3+^ into the cytosol enhances XoxF expression. A unique protein named lanmodulin from *Methylorubrum extorquens* strain AM1 was identified as a specific Ln^3+^-binding protein, and its biological function was implicated to be an Ln^3+^ shuttle in the periplasm. In contrast, it remains unclear how Ln^3+^ levels in the cells are maintained, because Ln^3+^ is potentially deleterious to cellular systems due to its strong affinity to phosphate ions. In this study, we investigated the function of a lanmodulin homolog in *Methylobacterium aquaticum* strain 22A. The expression of a gene encoding lanmodulin (*lanM*) was induced in response to the presence of La^3+^. A recombinant LanM underwent conformational change upon La^3+^ binding. Phenotypic analyses on *lanM* deletion mutant and overexpressing strains showed that LanM is not necessary for the wild-type and XoxF-dependent mutant’s methylotrophic growth. We found that *lanM* expression was regulated by MxcQE (a two-component regulator for MxaF) and TonB_Ln (a TonB-dependent receptor for Ln^3+^). The expression level of *mxcQE* was altered to be negatively dependent on Ln^3+^ concentration in ∆*lanM,* whereas it was constant in the wild type. Furthermore, when exposed to La^3+^, ∆*lanM* showed an aggregating phenotype, cell membrane impairment, La deposition in the periplasm evidenced by electron microscopy, differential expression of proteins involved in membrane integrity and phosphate starvation, and possibly lower La content in the membrane vesicle (MV) fractions. Taken together, we concluded that lanmodulin is involved in the complex regulation mechanism of MDHs and homeostasis of cellular Ln levels by facilitating transport and MV-mediated excretion.

## Introduction

*Methylobacterium* and *Methylorubrum* species are ubiquitous in nature and can be found in a variety of habitats, including soil, dust, freshwater, lake sediments, leaf surfaces, and nodules (Green and Bousfield, 1983). They belong to the commensal type 2 methylotrophs, which utilize single-carbon substrates such as methanol and other methylated compounds for assimilation *via* the serine cycle (Whittenbury and Dalton, 1981). As a predominant bacterial member on the aerial surface (phyllosphere) of plants, they are involved in the global cycle of single-carbon compounds, as well as in important plant health–mediating symbionts (Vorholt, 2012).

The initial crucial step for methylotrophy is methanol oxidation catalyzed by pyrroloquinoline quinone (PQQ) methanol dehydrogenases (MDHs) in *Methylobacterium* species. The classic calcium-dependent MDH known as MxaFI was believed to be essential for methylotrophic growth in the laboratory conditions (Chistoserdova et al., 2009). It was found that the presence of a lanthanide ion (Ln^3+^) strongly induces a homologous XoxF-type MDH in *Methylobacterium* species (Hibi et al., 2011), including a well-studied model strain *Methyorubrum extorquens* strain AM1 (Nakagawa et al., 2012). This discovery has attracted a great deal of attention, since it was the first demonstration of the involvement of Ln in biological molecular functions, and Ln had previously been believed to be biologically unnecessary. In addition, XoxF-type MDHs are more common in nature, ecologically more relevant, and older from an evolutionary perspective (Keltjens et al., 2014; Chistoserdova, 2016). A variety of bacteria exhibits methanol-oxidation ability or methanol growth only in the presence of Ln owing to the presence of XoxF and absence of MxaF (Fitriyanto et al., 2011; Pol et al., 2014; Lv et al., 2018, 2020; Wang et al., 2019; Wegner et al., 2019). *Methylobacterium extorquens* strain AM1 has two homologues of XoxF MDHs type I (XoxF1 and XoxF2) as well as Ln-dependent ethanol dehydrogenase (ExaF, Good et al., 2016).

Ln regulates the expression of these alcohol dehydrogenases in the organisms that possess both (“Ln-switch,” Vu et al., 2016; Masuda et al., 2018). *mxaF* expression is dependent on the presence of *xoxF* in addition to dual two-component regulatory systems, MxcQE and MxbDM. Further research found that Ln uptake into the cytosol, mediated by a system consisting of a TonB-dependent receptor (TBDR) and an ABC transporter, which is similar to the iron acquisition system. Once the Ln enters the cytosol it induces the expression of XoxF1-MDH in *M. extorquens* strain AM1 (Roszczenko-Jasińska et al., 2020) as well as in *M. extorquens* strain PA1 (Ochsner et al., 2019). The uptake system is encoded in an Ln utilization and transport (*lut*) gene cluster (META1_1778 to META1_1787) in strain AM1. Furthermore, a periplasmic protein encoded by META1_p1781 (*lutD*) in the *lut* cluster was shown to bind Ln^3+^ (Mattocks et al., 2019). In addition, an Ln^3+^-chelator biosynthetic gene cluster called Lanthanide Chelation Clusters (LCC, META1p4129 to META1p4138) in *M. extorquens* strain AM1, which encode a TBDR and non-ribosomal peptide synthetase (NRPS) enzymes, was reported. The NRPS proteins are similar to those for siderophore aerobactin synthesis, and chemically synthesized aerobactin was demonstrated to bind Ln^3+^, however, aerobactin does not have an effect *in vivo*, denoting that the product of LCC is not aerobactin. In addition, expression of LCC *in trans* promoted the bioaccumulation of Ln, but not Fe, indicating that the product of LCC is a novel Ln^3+^ chelator (lanthanophore, Zytnick et al., 2022). Thus, the Ln uptake mechanism consists of the lanthanophore, TBDR, ABC transporter, and many other uncharacterized proteins comprising a “lanthanome” in *Methylobacterium/Methylorubrum* species (Mattocks et al., 2019).

*Methylobacterium aquaticum* strain 22A is a plant growth–promoting bacterium isolated from a moss (Tani et al., 2011). The strain belongs to clade C in phenotypically heterologous genera *Methylobacterium/Methylorubrum* species (Green and Ardley, 2018; Alessa et al., 2021). The strain also has MxaF and XoxF as well as ExaF that are regulated by Ln, but its second XoxF (XoxF2) is a pseudogene (Masuda et al., 2018). We found that strain 22A also switches between *mxaF* and *xoxF* expression depending on the presence of La^3+^, and that several unknown genes were upregulated (Masuda et al., 2018). We found that a gene, Maq22A_c02050 within them, which encodes a protein annotated as “histidine kinase,” was upregulated by La^3+^. The amino acid sequence of the gene contains a signal peptide and EF-hand calcium-binding motifs, but not a kinase domain. Since the EF-hand motif is reported to bind Ln^3+^ (Ye et al., 2005), we are curious about the function of the protein.

This putative Ln^3+^-binding protein homolog was first characterized in *M. extorquens* strain AM1 as a high-affinity Ln^3+^-binding protein (Cotruvo et al., 2018; Cook et al., 2019). The protein was named lanmodulin (LanM) and its metal coordination motifs enable 100 million times more selective binding for Ln^3+^ and Y^3+^ than for Ca^2+^. The protein undergoes a metal-dependent conformational change. LanM may function as a periplasmic Ln-binding protein that acts in association with TBDR (Cotruvo et al., 2018; Chistoserdova, 2019). The LanM gene (*lanM*, META1_1786) is part of the *lut* cluster in strain AM1 and based on annotation, it locates in the periplasm, however, no phenotype has been found when lacking. The genetic knockout of a homolog (Mext_1854) in an *M. extorquens* strain PA1 ∆*mxaF* background did not result in a growth defect on methanol in the presence of La^3+^ (Ochsner et al., 2019). Thus, the function of LanM as an Ln^3+^-binding protein in the methylotrophy and physiology of *Methylobacterium* species is yet to be characterized. In this study, we performed a functional analysis of LanM in order to obtain clues on the involvement of this protein in the mechanism of Ln^3+^ transport and methylotrophy in strain 22A.

## Materials and Methods

### Strains and Culture Conditions

*Methylobacterium aquaticum* strain 22A (FERM-BP11078; Tani et al., 2012) was used in this study. Strain 22A was grown on R2A medium or mineral medium (MM; Alamgir et al., 2015) containing 0.5% methanol or 0.5% succinate, and both at 28°C. *Escherichia coli* DH5α was used for plasmid construction and *E. coli* S17-1 was used for conjugation; they were grown on LB medium at 37°C. Kanamycin (Km, 25 mg/l) and LaCl_3_ of various concentrations were added when necessary. We exclusively used plastics to prepare LaCl_3_-containing medium since La^3+^ binds to glass surfaces. For growth experiments, strain 22A and its derivatives were grown in 200 μl medium prepared in 96-well plates. To facilitate aeration, we bored 35 holes (Φ5 mm) between the wells on the bottom side, and removed the side skirts. In this condition, the plates were rotary-shaken at 300 rpm at 28°C. The growth was monitored by measuring OD_600_ using a microplate reader (PowerScan HT, DS Pharma).

### Recombinant LanM Expression and Purification

Alignment of protein sequences and signal peptide detection were done at EMBOSS water pairwise alignment^1^ and SignalIP-5.0 server,^2^ respectively. The strain 22A *lanM* (Maq22A_c02050) ORF encoding mature LanM without the signal peptide was PCR-generated using the primers listed in Supplementary Table S1. The product was cloned into the NdeI-XbaI site of pCold-I vector (Takara Bio Co.). The plasmid, pCold-*lanM*, was transformed into *E. coli* DH5α*. E. coli* DH5α (pCold-*lanM*) was cultured in 100 ml LB medium containing 25 mg/l ampicillin at 37°C until the culture OD_600_ became 0.5. Further cultivation was performed after the addition of 1 mM IPTG at 16°C for 18 h. The cells were collected, suspended in buffer A (20 mM Tris–HCl, pH 7.4, 70 mM NaCl and 20 mM imidazole), and disrupted with a bead beater (BioSpec 3110BX; Ieda Trading Corporation). The supernatant (centrifuged at 20,400 × *g* at 4°C for 15 min) was designated as a cell-free extract, and applied onto a Ni-NTA column (3.5 ml, His-Accept, Nacalai Tesque). The column was washed with 35 ml buffer A, and the His-tagged LanM (His-LanM) was eluted with buffer A containing 300 mM imidazole.

The ORF for mature *lanM* was also cloned into the *Bam*HI site of pGEX-6p-1 (GE Healthcare) to generate pGEX-*lanM*, and expressed as a GST-fusion protein (GST-LanM). DH5α (pGEX-*lanM*) was cultured in 100 ml 2xYT medium (16 g/l polypeptone, 10 g/l yeast extract, and 5 g/l NaCl) at 25°C until the culture OD_600_ became 0.7. Further cultivation was performed after the addition of 0.1 mM IPTG for 3 h. The cells were harvested, suspended in buffer B (20 mM Tris–HCl pH 7.5), and disrupted as above. The cell-free extract was applied onto a GST-ACCEPT column (1 ml, Nacalai Tesque). The column was washed with 10 ml buffer B, and the protein was eluted with buffer B containing PreScission protease (GE Healthcare).

The purified His-LanM and LanM (GST-cleaved) were buffer-exchanged to 40 mM acetate buffer, pH 4.1 containing 0.05% sodium azide with a 3 kDa cutoff filter (Amicon Ultra 3,000, Merck), and kept at 4°C until use. The concentration of protein was measured according to the Lowry method (Lowry et al., 1951) using bovine serum albumin as the standard.

### Biochemical Characterization of the Recombinant LanM

The purified His-LanM (86 μM) and LanM (43 μM) were subjected to gel filtration chromatography using 50 mM acetate-KOH buffer (pH 4.1) containing 100 mM NaCl and 0.05% sodium azide (TSK-GEL Super SW3000, 4.6 mm × 30 cm, flow rate 0.35 ml/min, injection 100 μl, detection 220 and 280 nm, fractionation 100 μl). The samples mixed with LaCl_3_ (final concentration 3.4 mM and 1.7 mM for His-LanM and LanM, respectively) were also analyzed. A fraction of the protein peak of LanM mixed with LaCl_3_ was selected (4.0–4.1 ml), mixed with nitric acid, and analyzed with inductively coupled plasma mass spectrometry (ICP-MS; Agilent Technologies 7500cx).

### Construction of Mutants

The gene deletion mutant of *lanM* was generated using an allele-replacement vector pK18mobSacB (Schäfer et al., 1994), as previously reported (Alamgir et al., 2015). In brief, each 1 kb upstream and downstream region of the target gene was polymerase chain reaction (PCR)-amplified and cloned in tandem into the EcoRI site of the vector and the In-Fusion Cloning kit (Takara Bio Co.). The vector was introduced into strain 22A and its derivatives by conjugation using *E. coli* S17-1. Single-crossover mutants were selected by Km resistance, and double-crossover mutants were selected by 10% sucrose resistance. PCR diagnosis was carried out as previously described (Alamgir et al., 2015). The *lanM* deletion mutant was designated as Δ*lanM*. Δ*mxaF*, Δ*xoxF1*, and Δ*xoxF1*Sup were generated in our previous study (Masuda et al., 2018). Δ*xoxF1*SupΔ*mxaF,* Δ*xoxF1*SupΔ*exaF*, and Δ*mxaF*Δ*exaF* were generated in this study using the gene deletion vectors made in our previous study (Yanpirat et al., 2020). In addition, we also constructed *ΔtonB_Ln* (Maq22A_c14845) and *ΔmxcQE* (Maq22A_c14840 and Maq22A_c14835).

### Vector Construction

We generated pCM130KmC for general cloning purposes that operates in strain 22A from pCM130 (Addgene plasmid #45828, Marx and Lidstrom, 2001) in our previous study (Yanpirat et al., 2020). A fragment containing an *Eco*RI site and His-tag coding sequence generated by a pair of complementary oligonucleotides (pCM130KmCinsert1 and 2, Supplementary Table S1) was inserted into the *Eco*RI site of pCM130KmC with the In-Fusion Cloning kit to generate pAT01. The vector enables His-tagged protein expression. Then we cloned PCR fragments of formaldehyde-activating enzyme promoter (P*_fae1_*, 220 bp upstream region of the *fae1* ORF, Maq22A_c16490) and *mxaF* promoter (P*_mxaF_,* 572 bp upstream region of the *mxaF* ORF, Maq22A_1p33165) into the *Eco*RI site, and named them pAT02f and pAT02m, respectively. These vectors enable His-tagged protein expression under P*_fae1_* and P*_mxaF_*. The PCR-amplified *lanM* ORF was cloned into pAT02f to express LanM, and introduced into Δ*lanM*.

To assess the promoter activity, PCR-generated green fluorescent protein (GFP) ORF from pHC42 (Chou et al., 2009) was cloned into each vector to generate pAT02f-GFP and pAT02m-GFP, using the primers listed in Supplementary Table S1. These vectors were introduced into strain 22A by conjugation and their fluorescence was measured to compare the expression level. The strains were grown on R2A medium containing 25 mg/l Km at 28°C for 3 days. The cells were harvested, washed with saline, and suspended in methanol medium at OD600 = 0.4. The suspension was aliquoted (200 μl) into black and transparent 96-well plates, and the cells were allowed to grow at 28°C, 300 rpm. The fluorescence (excitation 440/40, emission 528/20, sensitivity 120) and cell density (OD600) were measured with a microplate reader. The fluorescence was normalized to the cell density (OD600). The wild-type strain 22A harboring pAT02f-GFP or pAT02m-GFP showed higher GFP fluorescence when cultured on methanol than on succinate. The expression level of GFP in methanol in the absence of La^3+^ was higher than that in the presence of La^3+^ (Supplementary Figure S3). Based on these results, we used pAT02f as an expression vector for methanol-inducible *lanM* expression.

We constructed a GFP-tagged LanM expression vector based on pHC42 that has a GFP gene as a reporter. The ribosome binding site of the vector was eliminated by PCR with InversePHC42F2 and InversePHC42R2–2 primers and self-circularization of the product, and a new *Eco*RV site was concomitantly introduced. The vector was named pHC42m. Then, *lanM* with its putative promoter region (1,077 bp upstream region of the ORF) was PCR-amplified with lanM-promoter5’ and lanM-ORF3’ primers and cloned into the *Eco*RV site of pHC42m (pHC42m-*lanM*).

We also constructed a luciferase-reporter vector (pAT06-Lux) by replacing the P*_mxaF_* and GFP gene in pAT02m-GFP with bacterial luciferase (Lux) genes, which were PCR-amplified from pUC18-mini-Tn7T-Gm-lux (Wehrmann et al., 2017) with LuxC-F and LuxE-R2 primers. The promoter regions of *lanM* and *mxcQ* were PCR-amplified, and inserted into pAT06-Lux to generate pAT06-Lux-PlanM and pAT06-Lux-PmxcQ.

### qPCR

The total RNA was purified using Trizol (Sigma) from the exponentially growing culture of strain 22A wild type on methanol in the absence/presence of 30 μM LaCl_3_. The RNA samples were treated with Promega RQ DNase I (Promega). cDNA synthesis was carried out with ReverTra Ace (Toyobo) and a random hexamer primer. qPCR was performed with a CFX Connect Real-Time PCR detection system (Bio-Rad), Thunderbird SYBR green kit (Toyobo), and primers designed for quantitative (q) PCR (Supplementary Table S1). The thermal program was as follows: 95°C for 1 min, and 45 cycles of 95°C for 15 s and 62°C for 30 s, followed by dissociation curve analysis. The PCR-generated amplicon of the target region using the genome as a template was serially diluted and used for the generation of a standard curve. Data acquisition and analysis were performed with CFX Manager ver. 3.1 (Bio-Rad). The expression level of *lanM* was evaluated as a relative expression against *rpoC* (Maq22A_c27065), whose expression level is stable (Masuda et al., 2018).

### Luciferase Reporter Assay

Strain 22A and its derivatives transformed with pAT06-Lux derivatives were grown for 3 days at 28°C on R2A solid medium containing 25 mg/l Km. The cells were collected, washed with saline, and suspended in methanol medium, succinate medium, or methanol succinate medium at OD_600_ = 0.02. The medium suspension was aliquoted (200 μl) into 96-well white (for luminescence measurement, sensitivity 135) and transparent (for OD_600_ measurement) plates, and the plates were rotary-shaken at 28°C, 300 rpm. Promoter activity was evaluated as luminescence normalized to cell density (OD_600_). The expression level was regarded as the maximum luminescence value normalized by the OD_600_ value at that time.

### Observation of Cell Aggregation and Live/Dead Staining

The wild-type and Δ*lanM* cells grown on solid MM containing 0.5% methanol for 3 days were suspended in 10 mM HEPES, pH 7.0 (OD_60 0_ = 0.5). Then, LaCl_3_ was added (final concentration, 30 μM) and after 1 min, the cells were subjected to live/dead staining using a Viability/Cytotoxicity Assay kit for Bacteria (BTI Biotium Inc.) that contains DMAO (green fluorescent nucleic acid dye for staining live and dead bacteria) and ethidium homodimer (EthD-III, for staining dead bacteria). The cells were observed with a fluorescent microscope (Keyence BZ-X700). At the same time, the cell suspensions before/after 10 min addition of LaCl_3_ were serially diluted and spread onto R2A medium to count the number of colony forming units (CFUs).

### Cellular Uptake of La^3+^

The wild type, Δ*lanM*, and Δ*lanM* (pAT02f-*lanM*)(hereafter referred to as *lanM*-OX) grown on solid MM containing 0.5% methanol for 3 days were suspended in saline at OD_600_ = 0.3. The suspensions of 500 μl were aliquoted into 2 ml tubes and LaCl_3_ was added (final concentration, 30 μM). The suspensions were shaken (28°C, 160 rpm), and three tubes were centrifuged (20,400 × g, 4°C, 15 min) at the timing of 0, 4, 24, 48, 72, and 96 h. The supernatant (400 μl) was taken as a supernatant fraction. The cells and residual saline (100 μl) were mixed, transferred into a new tube, and designated as the precipitate fraction. Then the materials remaining on the wall of the emptied tubes were regarded as the tube wall fraction. The samples were mixed with 61% HNO_3_, heated at 100°C for 1 h, appropriately diluted, and analyzed with ICP-MS to measure the La content. The precipitate fraction was regarded as containing La associated with the cells and soluble La with an equal concentration as the supernatant fraction. Therefore, the soluble La content was subtracted from that of the precipitate fraction to give the cell-associated La content. As a control, saline without cells was prepared and treated in the same manner. The cell concentration of the samples before the addition of LaCl_3_ was determined by CFU measurement on R2A medium.

The tube wall fractions of 96 h samples prepared by another independent experiment were subjected to liquid chromatography (LC)-MS analysis. The tubes were washed twice with water, and vortexed after the addition of 1 ml acetonitrile. The contents were transferred into new tubes, centrifuged, dried *in vacuo*, dissolved in a buffer (8 M urea, 0.5 M Tris–HCl pH 8.5, and 25 mM EDTA), treated with 5 mg/ml DTT and 10 mg/ml iodoacetamide, and digested with trypsin. The samples were analyzed with a high performance LC-Chip/quantitative time of flight mass spectrometer (Agilent Technologies) at the Advanced Science Research Center, Okayama University, Okayama, Japan. The data were analyzed using Mascot (Matrix Science). The amino acid sequences of the CDSs extracted from strain 22A genome data served as the database.

### Confocal Laser Scanning Microscopy

Strain 22A Δ*lanM* transformed with pHC42m and pHC42m-*lanM* was grown in liquid MM containing 0.5% methanol in the absence/presence of 30 μM LaCl_3_ for 2 days, suspended in 5% glycerol, and observed with a confocal scanning microscope (FLUOVIEW FV1000, Olympus, ×100 objective lens) to observe the cellular localization of GFP-tagged LanM.

### Transmission Electron Microscopy

The wild type, Δ*lanM*, and *lanM*-OX grown in liquid MM containing 0.5% methanol in the absence/presence of 30 μM LaCl_3_ for 1 and 3 days were harvested, washed, and suspended in 2.5% glutaraldehyde in 0.1 M cacodylate buffer. The cells were washed with 0.1 M phosphate-buffered saline (PBS) four times, and mixed with 3% agarose. Then the samples were cut into 1 mm dice, fixed with 1.5% OsO_4_ in PBS for 1.5 h, washed with PBS three times, and dehydrated with ethanol (series of 50–100%, each 15 min). Ethanol was replaced with propylene oxide. The samples were embedded in Spurr resin (Polysciences), sliced with an ultramicrotome (Reichert-Jung), and stained with 3% uranyl acetate for 10 min and lead citrate for 2 min. Transmission electron microscopy (TEM) analysis was carried out with a JEM-1400 (JEOL, Ltd.). The electron-dense particles observed in the non-stained samples were also analyzed with a 200 kV TEM (JEM-2010) equipped with energy-dispersive X-ray spectroscopy (EDX; JED-2300, JEOL, Ltd.).

### Proteome Analysis for Δ*lanM*

The wild-type and Δ*lanM* cells grown in 100 ml MM containing 0.5% methanol in the absence/presence of 30 μM LaCl_3_ for 4 days in triplicate were harvested, washed, and suspended in 500 μl 8 M Urea containing 5 mM phenylmethylsulfonyl fluoride. The samples were processed similarly to the above for LC–MS analysis. The dried samples were dissolved in 50 μl 0.1% formic acid, and analyzed with TripleTOF^R^6600 (AB SCIEX, Tokyo Japan) equipped with Eksigent^™^ nanoLC. The Sequential Windowed Acquisition of all THeoretical fragment ions Mass Spectrometry (SWATH-MS) technique (Gillet et al., 2012) was utilized to identify and quantify the proteins. ProteinPilot software (Shilov et al., 2007) identified 18,061 peptides and 1,693 proteins (FDR 1%) through merge analysis of all samples, and they are used as a library for SWATH analysis. The data of peak area were normalized, and differentially expressed proteins were extracted under the following condition: fold change, 3 in comparison of the gene deletion effect (wild type against Δ*lanM*); *value of p* <0.05, pairwise-corrected *t*-test.

### MV Extraction and Proteome Analysis

Strain 22A wild-type (pAT02f), *ΔlanM*(pAT02f), and *lanM*-OX strains were grown in 30 ml of 0.5% methanol medium supplemented with 30 μM LaCl_3_ for 6 days with shaking at 28°C, 130 rpm in triplicate. The cultures were centrifuged twice at 5000 × *g* for 20 min at 4°C to remove bacterial cells, and then the supernatant was filtered through a 0.45 μm syringe filter. From these supernatants, Membrane Vesicles (MVs) were extracted according to the instructions in the ExoBacteria OMV Isolation Kit (SBI, code; EXOBAC100A-1). Of the 1.5 ml of each obtained MV extract, 400 μl was subjected to ICP-MS for quantification of La. The remaining extracts were used for protein concentration measurement by the BCA method and confocal microscopy. Each elemental data was normalized to the protein concentration of the respective MV extracts.

For proteome analysis of the MV fractions, the same strains were grown in 1 l medium similarly and the culture supernatant was filtered through a 0.45 μm syringe filter. These filtrates were ultracentrifuged (217,000 × *g*, 1 h, and 4°C, Beckman Coulter, Optima L-100 K), and the precipitated portions suspended in a buffer (12 mM sodium deoxycholate, 12 mM sodium N-lauroyl sarcosinate, 100 mM Tris–HCl pH 9.0; Masuda and Ishihara, 2016), and heated at 95°C for 5 min. The protein concentration was quantified using a BCA assay kit. DTT (final concentration 10 mM) was added to 300 μl of the samples. After 30 min, IAA (final concentration, 50 mM) was added. Then 1.2 ml of 50 mM ammonium hydrogen carbonate solution was added after 30 min incubation in the dark. For sodium N-lauroyl sarcosinate removal, 1.5 ml of ethyl acetate and TFA (final concentration 0.5%) were added, and the mixture was vortexed for 1 min. The upper ethyl acetate layer was removed by centrifugation. The remaining solution was dried *in vacuo*, and suspended in 100 μl of 5% ACN 0.5% TFA aqueous solution. The samples were desalted with C18-Tip, dried, and resuspended in 100 μl of 5% ACN 0.1% TFA for LC–MS analysis.

## Results

### LanM Sequence Characteristics and Its Upregulation in the Presence of La^3+^

*lanM* is encoded in the strain 22A chromosome, associated with the genes for purine biosynthesis, a hemolysin, a hypothetical protein, an adenylate cyclase, and an MucR transcriptional regulator (Supplementary Figure S2A). This approximately 4 kb region containing *lanM* is also conserved in the genomes of several *Methylobacterium* species, including *Methylobacterium* sp. strain 17Sr1-1 (CP029552.1), strain 17Sr1-28 (CP029553.1), and *M. currus* strain PR1016A (CP028843.1) that are phylogenetically close to strain 22A. The *lanM*s in strains AM1 and PA1 are clustered with *lut* genes related to ABC transporter and TBDR, which are important for Ln^3+^-dependent growth (Ochsner et al., 2019; Roszczenko-Jasińska et al., 2020). *lanM* in strain 22A appeared to be clustered with genes that are functionally unrelated to Ln transport.

The propeptide of LanM (134 amino acids) from strain 22A shares 58.5% identity and 68.9% similarity with that from strain AM1. A signal peptide cleavage site is detected at E23 or K25. Four repeats of the EF-hand motif (D(orE)xDxDxxxxxxE) were also detected. Unlike in the strain AM1 LanM holding proline at second amino acid of the four EF-hand motifs (Cotruvo et al., 2018), strain 22A LanM has T and K, respectively, in the first two motifs (Supplementary Figure S2B). These residues are not present in the structural homologs, calmodulin, and suggested to contribute to Ln selectivity.

**Figure 1:**
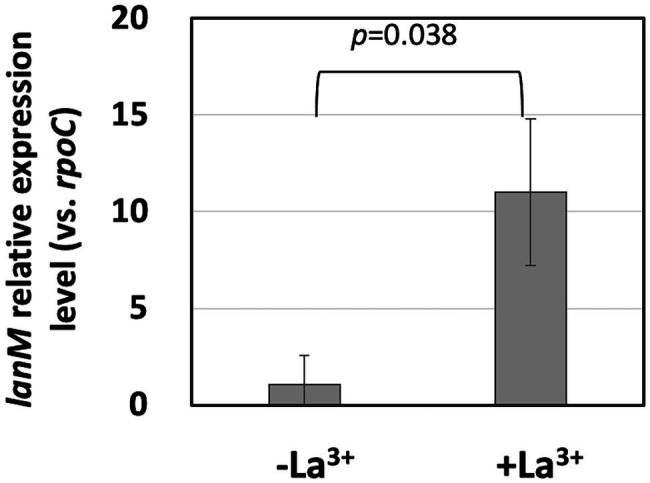
*lanM* expression level in *Methylobacterium aquaticum* strain 22A grown on methanol in the presence/absence of 30 μM La^3+^, quantified by qPCR. *lanM* expression level is shown as the relative expression level compared to that of *rpoC* as a reference. Data were analyzed with Student’s *t*-test.

In our RNA-sequencing experiment, *lanM* expression is 4.25-fold upregulated when the cells were grown on methanol in the presence of La^3+^, compared to the absence of La^3+^ (q < 0.001, two-way ANOVA followed by Tukey’s multiple comparison test, Masuda et al., 2018). We confirmed its upregulation with qPCR and found that the expression level is 10.3-fold upregulated in the presence of La^3+^, suggesting the importance of *lanM* for the growth in the presence of La^3+^ (Figure 1).

### LanM Binds La^3+^

**Figure 2:**
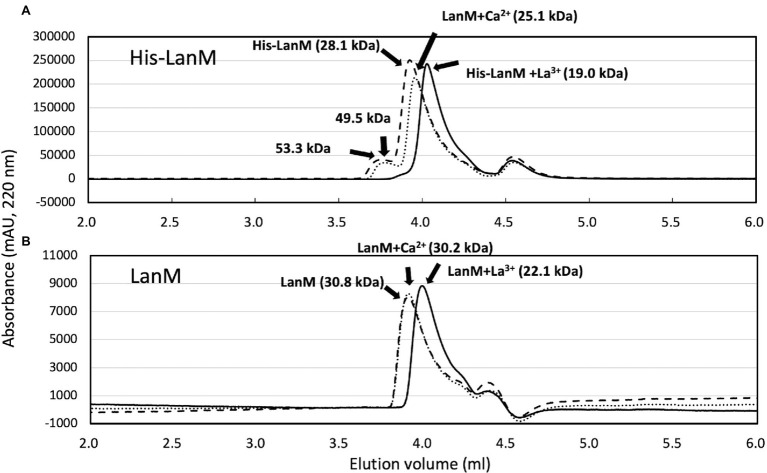
**(A)** Gel filtration analysis of His-LanM. The purified His-LanM (86 μM, 100 μl) was subjected to gel filtration analysis. Lines, absorbance at 220 nm; dashed line, His-LanM; solid line, His-LanM mixed with 3.4 mM LaCl_3_; and dotted line, His-LanM mixed with 3.4 mM CaCl_2_. **(B)** Gel filtration analysis of LanM. The purified LanM (43 μM, 80 μl) obtained by GST-cleavage of GST-LanM was mixed with 1.7 mM CaCl_2_ or 1.7 mM LaCl_3_ and subjected to gel filtration. Lines, absorbance at 220 nm; dashed line, His-LanM; solid line, His-LanM mixed with La^3+^; and dotted line, His-LanM mixed with Ca^2+^. Protein molecular weight analysis was carried out with separately analyzed standard proteins of chicken egg albumin, 45 kDa; bovine milk α-lactalbumin, 14.2 kDa; cytochrome c 12.4 kDa; and aprotinin 6,511 Da.

To biochemically characterize LanM from *M. aquaticum* strain 22A, we purified the recombinant mature LanM (E23 was regarded as the cleaving site, and its theoretical molecular mass is 11.85 kDa) expressed in *E. coli* as an N-terminal His-tagged protein (His-LanM, theoretical mass 14.0 kDa) and N-terminal GST-tagged protein (GST-LanM, 36.7 kDa; Supplementary Figure S3). The GST-LanM was cleaved to remove the GST portion (LanM, 12.3 kDa). The purified His-LanM and LanM were mixed with La^3+^ or Ca^2+^ and analyzed with gel filtration chromatography (Figure 2). The apparent native molecular mass was 28.1 kDa (His-LanM) and 30.8 kDa (LanM), which shifted to 19.0 kDa and 22.1 kDa when mixed with La^3+^, respectively. That of His-LanM mixed with Ca^2+^ also shifted to 25.1 kDa. The His-LanM and that with Ca^2+^ showed an additional peak at 53.3 and 49.5 kDa, respectively, which disappeared upon the addition of La^3+^. These higher molecular peaks did not appear in the LanM preparation. The peak fraction of LanM (4.0–4.1 ml) mixed with La^3+^ was analyzed with ICP-MS. The protein concentration was 25.3 μM and the La^3+^ content was 92.5 μM, suggesting that LanM binds 3.65 mol La^3+^ per LanM molecule. La^3+^ was not detected in the experiment without LanM and that without added La^3+^, at the fraction of the same elution volume (Data not shown). These results suggested that LanM alters its conformation to a more compact form upon binding La^3+^, as already shown for the strain AM1 LanM (Cotruvo et al., 2018; Cook et al., 2019).

### LanM Is Not Essential for Methanol Growth

**Figure 3:**
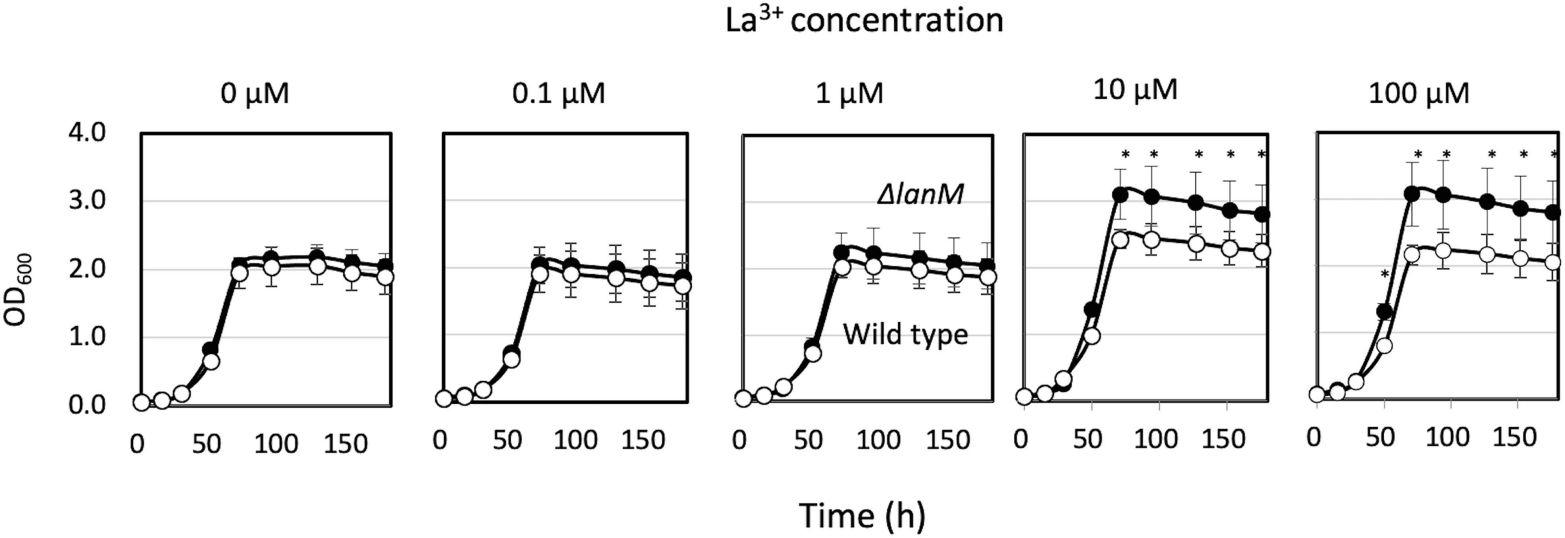
Growth of strain 22A wild type and Δ*lanM* on methanol under varied concentrations of LaCl_3_. Filled symbols, wild type; open symbols, Δ*lanM*. Data are presented as the mean value ± standard deviation (SD; *n* = 3). The data were analyzed with two-way ANOVA followed by Sidak’s multiple comparisons test. ^*^p < 0.05.

**Figure 4:**
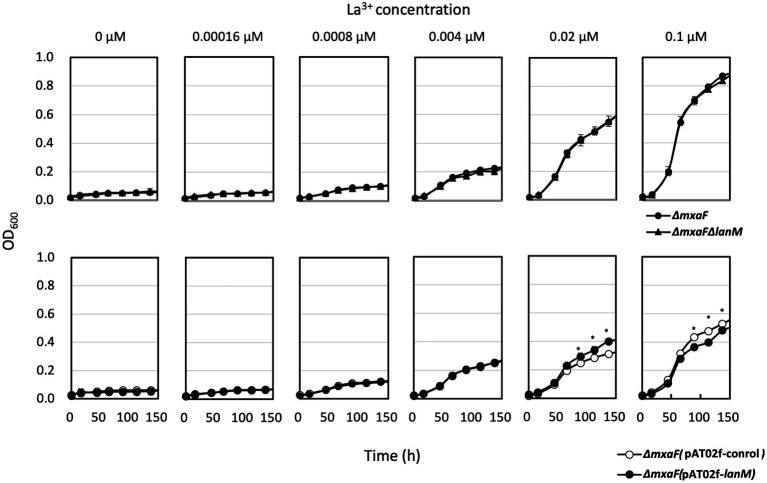
Upper panel: Growth of Δ*mxaF* (filled circles) and Δ*mxaF*Δ*lanM* (triangles) on methanol in the presence of limited concentrations of La^3+^. Lower panel: Growth of Δ*mxaF* (pAT02f; open circles) and Δ*mxaF* (pAT02f-lanM; filled circles) on methanol in the presence of limited concentrations of La^3+^. Data are presented as the mean value ± standard deviation (SD; *n* = 3). The data were analyzed with two-way ANOVA followed by Sidak’s multiple comparisons test. ^*^p < 0.05.

Δ*lanM* did not show any growth deficiency on methanol in the presence of varied concentrations of La^3+^, but showed a higher cell yield than the wild type grown with relatively high La^3+^ concentrations (10–100 μM; Figure 3). These results suggested that LanM is not required for methanol growth. Next, Ln^3+^- and XoxF-dependent methanol growth of *ΔmxaF* was examined in the presence of limited La^3+^ (Figure 4). *ΔmxaFΔlanM* showed no difference in La^3+^ concentration–dependent growth compared to *ΔmxaF*, suggesting that LanM is not essential for XoxF expression and XoxF metalation. When *lanM* was expressed under the *fae1* promoter, the strain exhibited slightly faster (with 0.02 μM La^3+^) and slightly slower (with 0.1 μM La^3+^) growth. These results suggested that overexpression of LanM causes some unknown dysregulation of metabolism.

### Identification of an Ln^3+^-Transporting TBDR

Among 12 TBDR genes encoded in the genome of strain 22A, Maq22A_c14845 showed the highest identity to *lutH* in the *lut* gene cluster of strain AM1 (MexAM1_META1p1785, 35.7% identity, EMBOSS water pairwise alignment). The expression of Maq22A_c14845 was induced 1.3-fold by La^3+^ (Masuda et al., 2018). Th gene is encoded in the upstream of *mxcQE* in the same orientation, but there are no other apparent methylotrophy- or lanthanide uptake-related genes nearby. Knockout strains of Maq22A_c14845 were successfully generated under wild-type and *ΔmxaF* backgrounds, and subjected to growth experiments on methanol and succinate. The gene was named *tonB_Ln.*

*ΔtonB_Ln* showed no growth deficiency on methanol irrespective of the presence of La^3+^, because the mutant could use MxaF to grow even in the presence of La^3+^ (Supplementary Figure S4). Whereas *ΔmxaF* could still grow in the presence of La^3+^ but not in the absence of La^3+^, *ΔmxaFΔtonB_Ln* could not grow in the presence of La^3+^. These results suggested that TonB_Ln is involved in Ln^3+^ uptake that leads to the activation of XoxF. Their growth on succinate was not affected by La^3+^.

### *lanM* Expression Is Regulated by *mxcQE* and *tonB_Ln*, and *mxcQE* Expression Is Regulated by *lanM*

**Figure 5:**
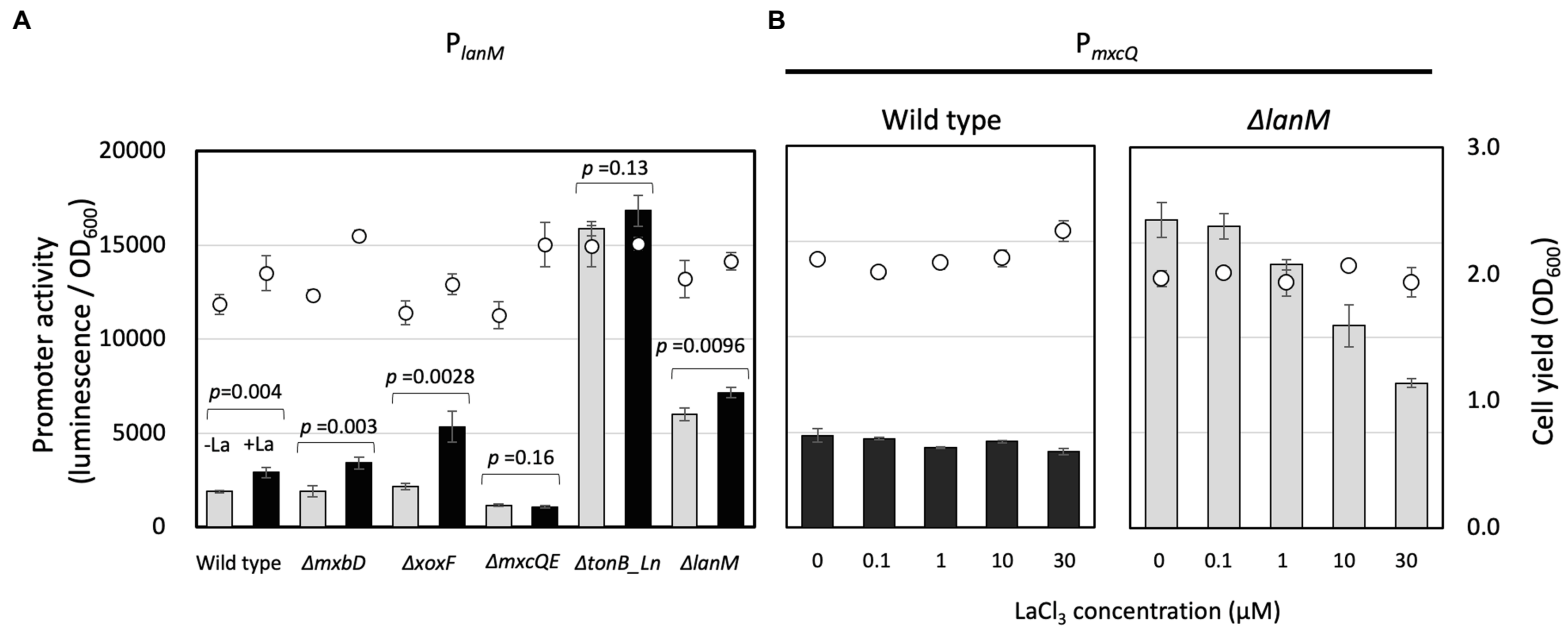
**(A)** P*_lanM_* activity in the mutants grown on methanol plus succinate. Promoter activity was assessed as maximum luminescence per OD600 of the culture at the time. Gray bars, without LaCl_3_; filled bars, with 30 μM LaCl_3_; circles, cell yield (OD600). The promoter activity was analyzed by Student’s *t*-test and *p* values are indicated. **(B)** P*_mxcQ_* activity in the wild type and Δ*lanM* grown on methanol in the presence of varied concentrations of LaCl_3_. Bars, P*_mxcQ_* activity; and circles, cell yield (OD600). Data are presented as the mean value ± standard deviation (SD; *n* = 3).

To examine genes involved in the regulation of *lanM,* the *lanM* promoter reporter vector (pAT06-Lux-P*lanM*) was introduced into several mutants of strain 22A (Figure 5A). The transformants were grown on succinate and methanol. In the wild type and ∆*mxbD*, P*_lanM_* responded to the presence of La^3+^. In *ΔxoxF*, P*_lanM_* responded to La^3+^ even higher than the wild type. In *ΔmxcQE* and *ΔtonB_Ln,* P*_lanM_* activity was lower and higher than that in the wild type, respectively, and no La^3+^-induction was observed. In *ΔlanM*, interestingly, P*_lanM_* was highly active even in the absence of La^3+^. These results suggested that P*_lanM_* is under regulation of *mxcQE* and *tonB_Ln*, and that P*_lanM_* shows self-regulation, as seen in the case of P*_xoxF_*.

*P_mxcQ_* showed little response to La^3+^ in the wild-type background, however, it showed higher activity than in the wild type, and it responded negatively to La^3+^ concentration in the *ΔlanM* background (Figure 5B), suggesting that LanM is involved in the Ln^3+^ concentration–dependent regulation of *mxcQ*.

### Cell Membrane Integrity Was Impaired in *ΔlanM*

**Figure 6:**
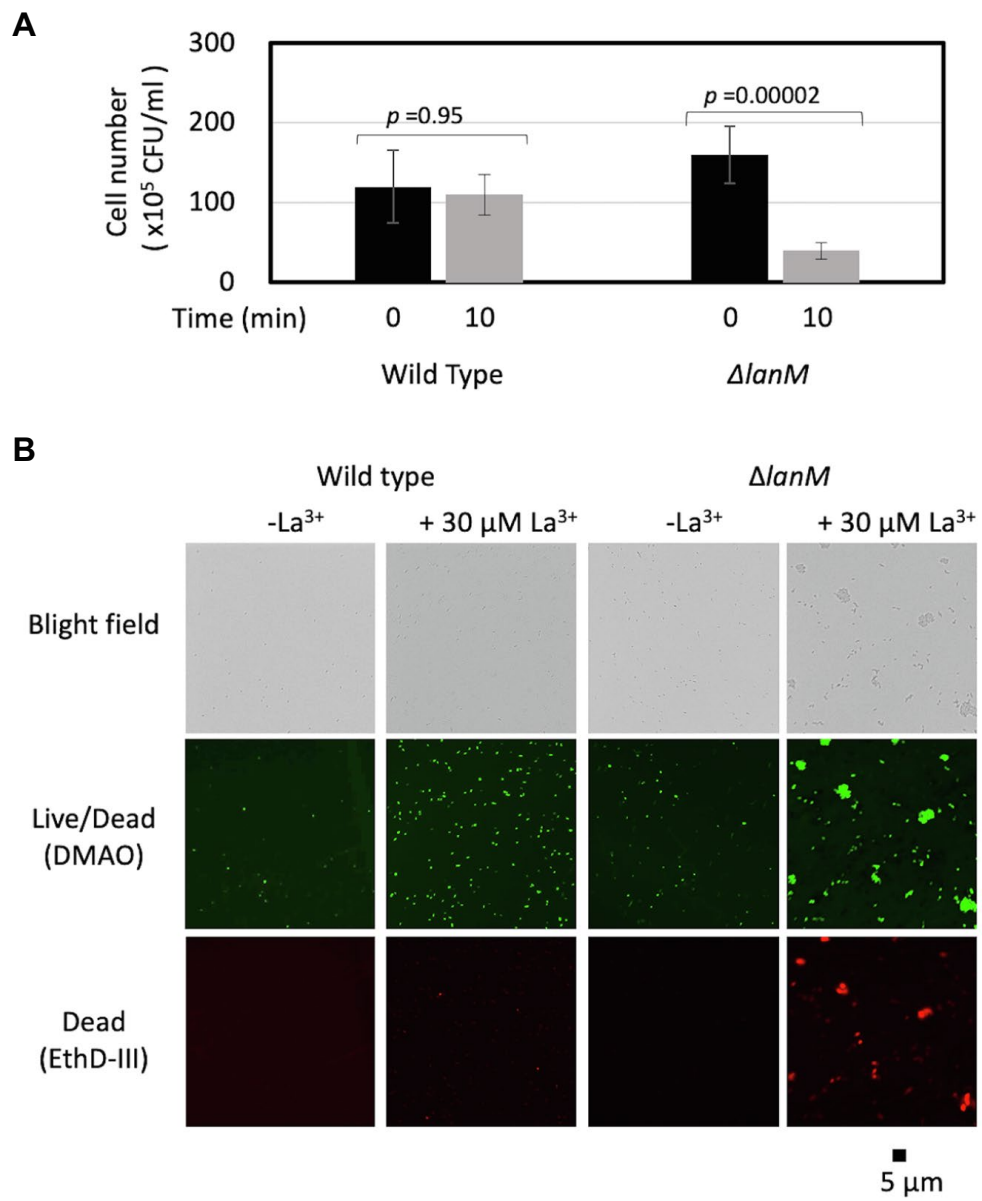
Cell aggregation of Δ*lanM* upon exposure to La^3+^. **(A)** Determination of CFUs of the wild type and Δ*lanM* before and after exposure to 30 μM La^3+^. Data are presented as the mean value ± standard deviation (SD; *n* = 3) and analyzed with Student’s *t*-test. **(B)** Fluorescent microscopic analysis of the Live/Dead-stained wild type and Δ*lanM* exposed to 30 μM La^3+^.

We noticed that Δ*lanM* cells tend to aggregate upon the addition of La^3+^ in a 10 mM HEPES buffer (pH 7.0). We therefore assessed the viability of Δ*lanM* upon exposure to La^3+^. We found that the CFUs of Δ*lanM* decreased upon the addition of La^3+^ in 10 min (Figure 6A). The Δ*lanM* cells aggregated in the presence of La^3+^ and showed high permeability for ethidium homodimer III (EthD-III), which is used to assess cell death (Figure 6B). In physiological saline, this phenomenon did not occur (data not shown); therefore, this effect was dependent on HEPES. Since HEPES is known to be cytotoxic (Zigler et al., 1985), these results suggested that the cell membrane integrity is impaired in Δ*lanM* upon exposure to La^3+^.

### Uptake of La^3+^

**Figure 7:**
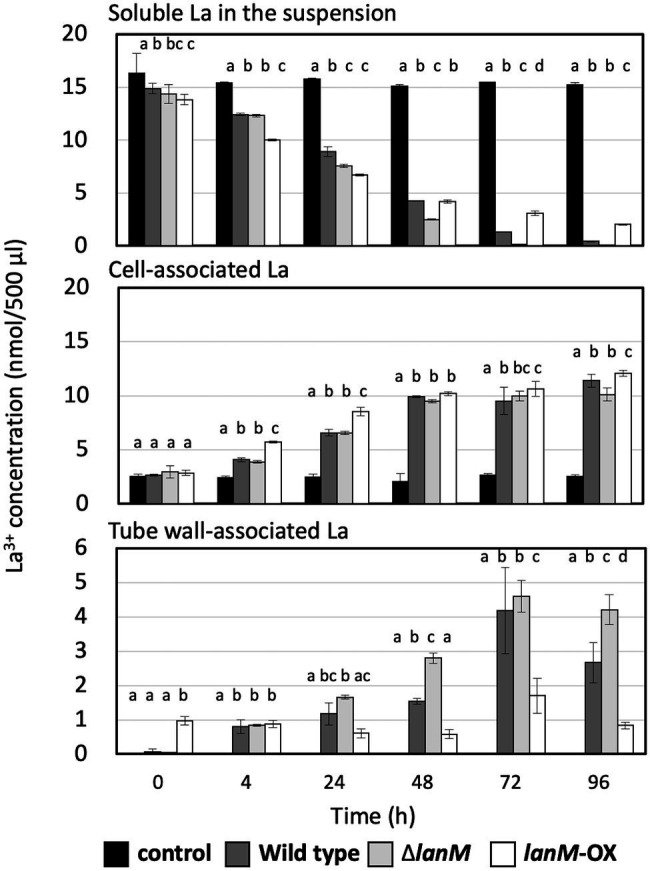
Time-dependent changes of the soluble La in the suspension, cell-associated La, and tube wall–associated La in the cell suspensions of the wild type, Δ*lanM*, and Δ*lanM* (pAT01-P*fae1*-*lanM*). The details of the experiment are included in the “Materials and Methods”. Data are presented as the mean value ± standard deviation (SD), two-way ANOVA with a Tukey’s multiple comparisons test and compact letter display (*p* < 0.05, *n* = 3).

Next, we examined the time-dependent Ln^3+^ uptake in the wild type, Δ*lanM*, and *lanM*-OX in the presence of 30 μM La^3+^ (Figure 7). The cell concentration of the suspensions was 39.3 ± 2.5 × 10^6^ CFUs (wild type, data presented as mean ± standard deviation [SD], technical triplicates), 42.7 ± 2.3 × 10^6^ CFUs (Δ*lanM*), and 52.3 ± 3.1 × 10^6^ CFUs (*lanM*-OX). The La^3+^ content in three fractions, the supernatant of the cell suspension, the cells, and the remnants on the tube wall, were measured. The control experiment without the cells showed almost constant La^3+^ concentration in the supernatant and the precipitate, and La^3+^ absorption to the tube wall was not observed. The La^3+^ concentration in the supernatant of the wild-type cell suspension decreased over time, down to almost zero in 96 h, and the cell-associated La^3+^ content became greater. A substantial amount of La^3+^ was also detected in the tube wall fraction. Δ*lanM* showed a similar trend but the decrease in supernatant La^3+^ was faster, and the La^3+^ content in the tube wall fractions was greater than the wild type, whereas it showed comparative La^3+^ content in the cell-associated fraction. *lanM*-OX showed an even faster decrease in the supernatant within 24 h, but later the supernatant La^3+^ content became higher than the other two. Whereas the La^3+^ content in the cell-associated fraction was higher in the earlier timing (4–24 h), the tube wall fraction contained the least La in the end. These results suggested that the LanM facilitates Ln^3+^ uptake, and that the lack of LanM causes aggregation Ln^3+^-bound cells.

We were interested in the La^3+^ found in the tube wall fraction. Another independent experiment in the same setting was conducted, and the 96 h samples of tube wall fractions were subjected to tryptic digestion and proteomics analysis. As shown in Supplementary Table S2, the only protein detected in the samples of the wild-type and *lanM*-overexpressing strains was flagellin. Various major proteins, including Fae1, MtdA, and PhaA, in addition to the flagellin were detected in the Δ*lanM* sample. Due to the intracellular nature of some of these proteins, these results suggested that the Δ*lanM* cells adhered to the tube wall in the presence of La^3+^, possibly due to damage or disintegration of the cell outer membrane, which did not happen in the wild-type and *lanM*-overexpressing strain. We also could not rule out possible cell lysis and resultant adhesion of proteins.

### Subcellular Localization of LanM and La Deposition in the Cells

**Figure 8:**
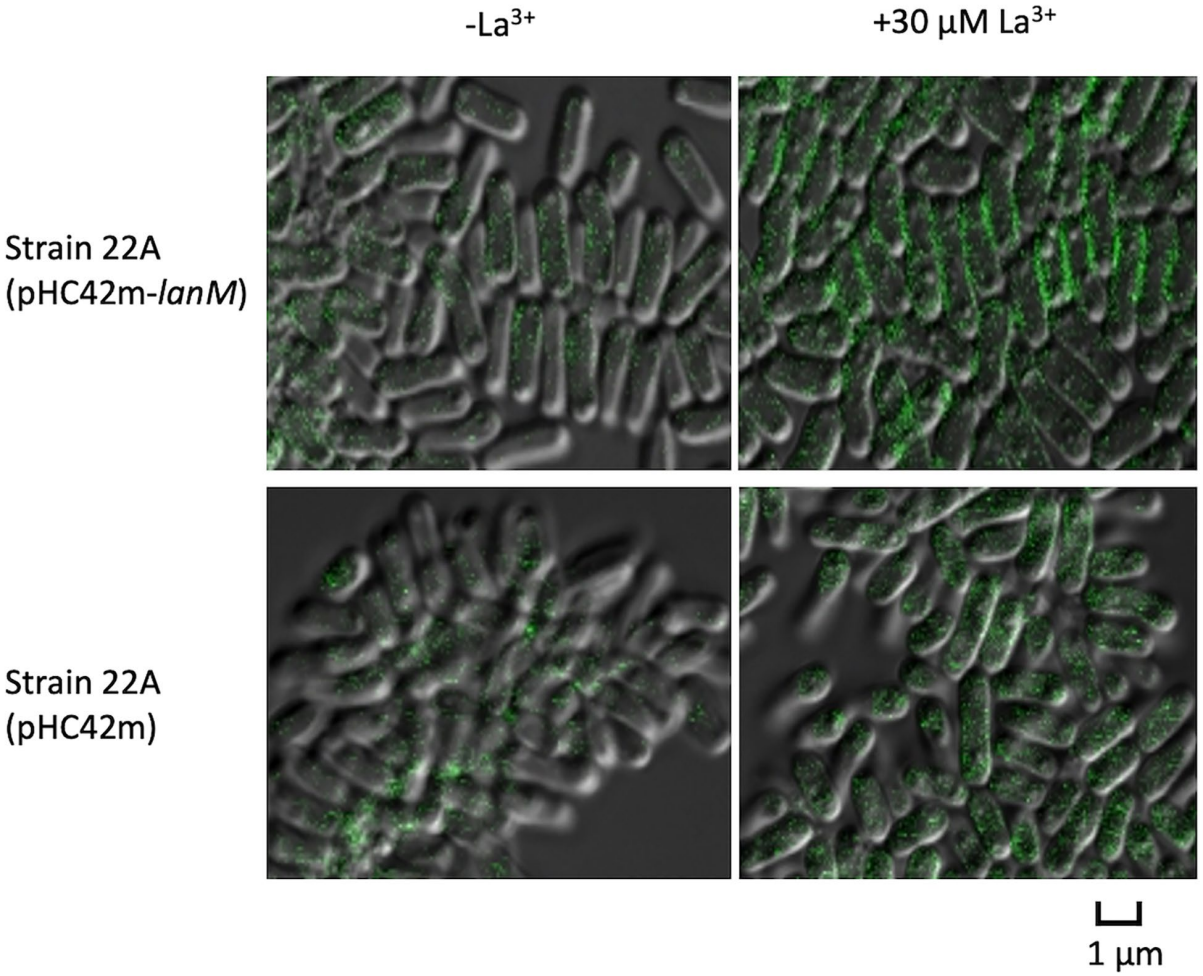
Cellular localization of GFP-tagged LanM in strain 22A cells. Strain 22A wild type containing pHC42m-*lanM* and pHC42m was grown on methanol in the absence/presence of 30 μM La^3+^ for 3 days and observed with confocal fluorescence microscopy. Bar, 1 μm.

We next examined the subcellular localization of GFP-tagged LanM in Δ*lanM*. The GFP signal was found mainly in the periphery of the cells, and it was brighter in the presence of La^3+^ (Figure 8). The cells expressing only GFP showed intracellular GFP signals irrespective of La^3+^. With the presence of the signal peptide (Supplementary Figure S1B), this result suggested that LanM is a periplasmic protein.

**Figure 9:**
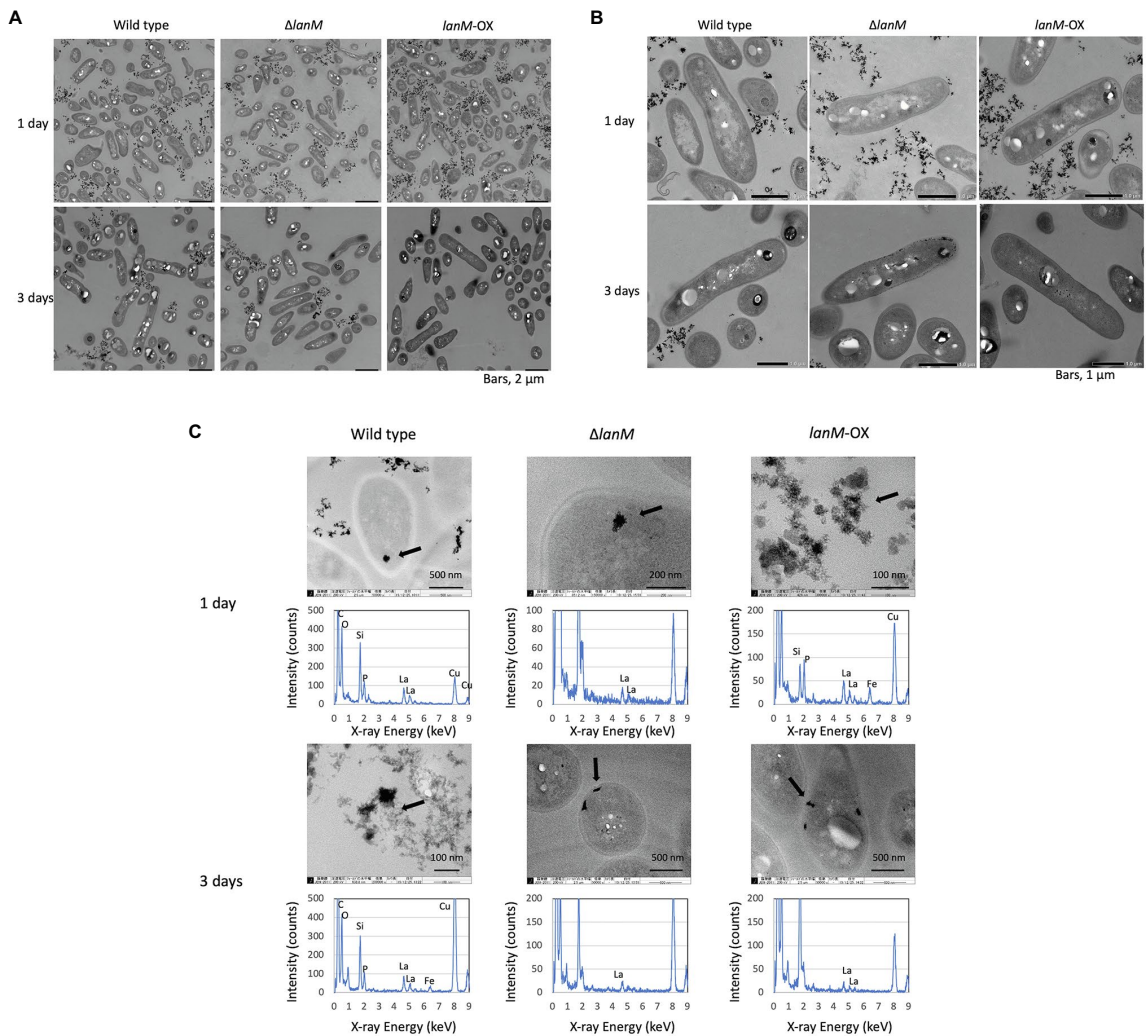
TEM images of wild type, Δ*lanM*, and Δ*lanM* (pAT01-P*fae1*-*lanM*) grown in liquid mineral medium containing 0.5% methanol and 30 μM La^3+^. **(A,B)** Cells stained with uranyl acetate and lead citrate. **(C)** EDX analysis for electron-dense particles observed in each sample. Arrows indicate the EDX-analyzed positions. The detected Si and Cu are derived from TEM lens tube and grids, respectively.

Next, we observed the cells of the wild type, Δ*lanM*, and *lanM*-OX with a TEM (Figure 9A). The medium contained insoluble deposits in all samples, and the amount became smaller in 3 days than in 1 day, suggesting that the deposits were solubilized according to the cell growth. They were mixtures of at least two different substances (shown in the wild type, 3 day culture, and *lanM*-OX, 1 day culture); the denser particles were found to contain La, P, and O, and less dense particles were found to contain Fe, P, and O. Therefore, the La and Fe added in the medium was assumed to form insoluble particles with phosphates in our medium. Occasionally, electron-dense particles sized 0.1 to 0.2 μm could be observed in the cells of all samples, especially in the 3 day samples. We found that Δ*lanM* cells showed electron-dense deposits in the periplasm (Figure 9B). In the non-stained samples, we found electron-dense deposits in all cell samples; a typical one in the wild type (1 day) is shown in Figure 9C. EDX analysis showed that they contain La, P and O, whereas a part of cells that apparently not containing the deposit did not show any of these elements (data not shown). Occasionally, cells exhibited specific La-containing relatively large deposits at the periplasm. Typical ones in Δ*lanM* and *lanM*-OX are shown. Similar deposits have been also observed in *M. extorquens* strain AM1 *lut* gene mutants (Roszczenko-Jasińska et al., 2020) and *Beijerinckiaceae* bacterium RH AL1 (Wegner et al., 2021).

### Proteome Analysis for Δ*lanM*

Through SWATH-MS proteome analysis of the wild type and Δ*lanM*, we could quantify 1,263 proteins and 3,597 peptides in total (Supplementary Table S3). The differentially expressed proteins were extracted and are shown as a heatmap in Supplementary Figure S5. There was no protein involved directly in methylotrophy, which is understandable because Δ*lanM* did not show any defects in methanol growth. Maq22A_c16420 encoding an ABC transporter is most homologous to LutE (MexAM1_META1p1782), recently identified in *M. extorquens* strain AM1 as a component for an Ln^3+^ transporter across the inner membrane. The expression of the protein is upregulated in the wild type but repressed in Δ*lanM* in response to La^3+^, suggesting that *lanM* deletion resulted in restricted Ln^3+^ uptake into the cell. Transmembrane glycoprotein N-acetylglucosamine-1-phosphodiester-alpha-N-acetylglucosaminidase (Maq22A_c12225), UDP-diphospho-muramoylpentapeptide-beta-N-acetylglucosaminyltransferase (Maq22A_c12405), cell wall metabolism sensor kinase (Maq22A_1p36015), and D-alanyl-D-alanine carboxypeptidase (Maq22A_c17650) were differentially expressed together with fatty acid biosynthesis genes such as biotin carboxyl carrier protein (Maq22A_c14865) and 3-hydroxyacyl-CoA dehydrogenase (Maq22A_2p41960), suggesting that cell wall biogenesis is differentially regulated in Δ*lanM*. In addition, the differentially expressed inositol monophosphatase (Maq22A_c12285) and phosphate starvation protein PhoH (Maq22A_1p32125) suggested altered phosphate metabolism in the cell, as Ln^3+^ is known to bind phosphate. MxaK protein is involved in Ca^2+^ insertion into MxaF (Richardson and Anthony, 1992), which was also differentially regulated in Δ*lanM.*

### MV Analysis

Confocal microscopy images of MVs extracted by the OMV Extraction Kit from the cultures of wild-type, *ΔlanM*, and *lanM*-OX grown in methanol medium supplemented with 30 μM LaCl_3_ showed that MVs from *lanM*-OX appeared darker than those from the others (Supplementary Figure S6). MVs were scarce in the *ΔlanM* culture. The MV fraction from *LanM-OX* collected by ultracentrifugation also showed more blackish particles compared to those from wild type and *ΔlanM*.

The La content of the kit-purified MV fractions was analyzed by ICP-MS. The amount of La normalized to the amount of protein revealed that LanM-OX showed the highest La content, although there were no statistically significant differences due to high variance (Supplementary Figure S7).

Next, the ultracentrifugation-purified MV fractions were subjected to LC–MS analysis. The detected proteins are summarized in Supplementary Table S4. The protein concentrations of the samples for LC–MS analysis were 1.2, 0.37, and 0.45 mg/ml for the wild type, *ΔlanM*, and LanM-OX, respectively. The presence of a number of outer membrane proteins, including porin and flagella proteins, indicated that the MV fractions indeed contained MVs. The sample from the wild type contained many cytoplasmic proteins, including those involved in methylotrophy, which might suggest some contamination of the cells or abundant proteins in the cells. The fractions from *ΔlanM* and *lanM-OX* contained some unique proteins, which are involved in flagella, porin, and an unknown outer membrane protein. Through this qualitative analysis, we did not identify any proteins that are apparently or possibly involved in Ln sequestration, however, it is of note that LanM protein and all other periplasmic proteins, such as MDHs, were not detected in the samples.

## Discussion

In this study, we focused on LanM in strain 22A and attempted to determine its biological and physiological function. In our previous study, we predicted that the protein would bind Ln^3+^ (Masuda et al., 2018). Actually, LanM from *M. extorquens* strain AM1 was shown to bind Ln^3+^ (Cotruvo et al., 2018; Cook et al., 2019). However, no phenotype was observed in *M. extorquens* strain PA1 (Ochsner et al., 2019) and the screen-based on transposon mutagenesis did not identify *lanM* as a gene encoding a product essential for lanthanide-dependent growth (Roszczenko-Jasińska et al., 2020). It has been suggested that the protein shuttles Ln to Ln^3+^-dependent enzymes (Cotruvo et al., 2018; Cook et al., 2019). LanM also responds to Ln^3+^ in non-methylotrophy settings in a methylotrophic *Beijerinckiaceae* bacterium RH CH11 (Wegner et al., 2021).

*lanM* is associated with other *lut* genes in strain AM1 and PA1 genomes, but it is associated with the genes of different functions in strain 22A (Supplementary Figure S2). The amino acid sequence of TlyC (hemolytic element) includes a transmembrane domain (DUF21, pfam01595), a CBS_pair_CorC_HlyC_assoc (cd04590) domain, and a transporter-related domain (CorC_HlyC, smart01091). The latter two domains are related to magnesium and cobalt efflux. A hypothetical protein gene (c02045) adjacent to *lanM* has no known domains. Next, the gene encodes an adenylate cyclase that is a eukaryotic protein activated by calmodulin, to which LanM shows homology. In the case of pathogenic *Bordetella pertussis*, adenylate cyclase increases cAMP levels in the human host in response to host calmodulin (Voegele et al., 2018). Transcriptional regulators of the MucR family are involved in extracellular polysaccharide synthesis in *Rhizobium* species (UniProtKB-P55323). At the moment, it is unknown whether these gene products are associated with LanM function, but similar gene clusters are conserved in phylogenetically related species of *Methylobacterium,* suggesting their possible functional association.

The increased expression of *lanM* in response to La^3+^ (Masuda et al., 2018) was confirmed in this study (Figure 1). The conformational change upon binding of La^3+^ (Figure 2) is likely important for its interaction with other proteins. Regardless of La^3+^, LanM is not required for the wild type to grow on methanol, and growth of the *lanM* mutant is rather better than that for the wild type (Figure 3). LanM is not essential for XoxF expression and XoxF metalation (Figure 4). These data suggested that LanM is not necessary for the methylotrophy in strain 22A, despite its Ln^3+^-binding ability.

Ln^3+^ is transported into the periplasm by a TBDR, one of which has been identified in strain AM1. We found the most homologous TBDR gene and generated a knockout mutant, accompanied with *mxcQE*, which is involved in MDH regulation. We confirmed that *tonB_Ln* is necessary for XoxF-dependent methanol growth and that *mxcQE* is necessary for MxaF-dependent methanol growth (Supplementary Figure S4). P*_lanM_* is regulated by the *mxcQE* and *tonB_Ln*, and P*_lanM_* exhibits self-regulation, as observed in the case of P*_xoxF_* (Figure 5). In addition, P*_mxcQ_* showed a constant expression level independent of LaCl_3_ concentration in the wild type, whereas it showed an La-dependently decreasing expression level in ∆*lanM* (Figure 5). These results suggested that there is a system monitoring Ln levels through the lanM function. In the wild type, MxcQE expression is maintained constant regardless of the presence of Ln^3+^, which is controlled by LanM. Because MxcQE is responsible for MxaF expression, LanM is partly involved in the Ln-switch, although there was little change in the methylotrophic phenotype. The full picture of the Ln-switch mechanism is currently patchy, but here we can add LanM as one of the important players involved in the regulation. To clarify this complex mechanism, it is required to identify the ligands for, and the genes regulated by, the dual two-component signaling systems of MxcQE and MxbDM.

*ΔlanM* showed an obvious increase in cell aggregation and membrane permeability upon exposure to La^3+^ (Figure 6). These results suggested that *ΔlanM* altered the integrity of the cell membrane. Since *ΔlanM* did not exhibit growth retardation (the cell yield increased in the presence of high La^3+^, Figure 3), this altered membrane integrity was not detrimental to cells, but was rather temporal. The aggregation might suggest neutralization of the cell-surface negative charge by La^3+^, which would result in the cell adhesion to plastic tubes (Supplementary Table S2). LanM overexpressing strain showed faster La^3+^ uptake and subsequently higher amounts of extracellular soluble La (Figure 7). LanM localized to the periplasm (Figure 8), and *ΔlanM* showed La deposition in the periplasm (Figure 9). These results suggested that LanM simultaneously enhances both La^3+^ uptake and the efflux of soluble La^3+^ in the form of MV (Supplementary Figures S5–S7). Since LanM was not detected in the MV proteomics samples (Supplementary Table S4), there may be other proteins or factors that selectively translocate Ln (deposition) into the MV. Regulation of MV synthesis and selection of proteins into MV cargo are now an emerging field of microbiological studies (Toyofuku et al., 2018). MV formation has been suggested as one of the Ln uptake systems in the methylotrophic *Beijerinckiaceae* bacterium RH AL1 (Wegner et al., 2021). MV is also reported to be involved in the excretion of excess copper (Lima et al., 2022), suggesting that MV formation is one of the cellular strategies to excrete excess metals to maintain metal homeostasis. PQQ can bind Ln^3+^ (Lumpe and Daumann, 2019) as a possible factor for Ln excretion. PQQ is secreted to medium when strain 22A grows on methanol in the absence of La^3+^, but the presence of La^3+^ represses it (Masuda et al., 2018); this was also found in strain AM1 (Good et al., 2016). Thus, PQQ is also suggested to be one of the lanthanophores (Good et al., 2022), however, it is unknown whether PQQ is compartmented with Ln in the MV. The lanthanophore biosynthesis genes found in *M. extorquens* strain AM1 are similar to those of aerobactin siderophores (Zytnick et al., 2022). Such lanthanophores may work in Ln compartmentation into the MV. Thus, there is much to be investigated to fully understand the roles of these factors in Ln uptake and excretion. Taken together, we conclude that LanM maintains the Ln level in the periplasm by facilitating the uptake as well as excretion into MV, and this Ln-responsive process is regulated by MxcQE and the Ln-transporting TBDR, comprising a part of lanthanome and the Ln-switch.

## Data Availability Statement

The original contributions presented in the study are included in the article/Supplementary Material, further inquiries can be directed to the corresponding author.

## Author Contributions

YF and AT designed the research, performed the experiments, performed the data analysis, and drafted the manuscript. TS analyzed proteomics samples. All authors reviewed the manuscript. All authors contributed to the article and approved the submitted version.

## Funding

This work was supported in part by MEXT KAKENHI (18H02129 and 21H02105 to AT). The funders had no role in study design, data collection and interpretation, or the decision to submit the work for publication.

## Conflict of Interest

TS was employed by K.K. AB SCIEX.

The remaining authors declare that the research was conducted in the absence of any commercial or financial relationships that could be construed as a potential conflict of interest.

## Publisher’s Note

All claims expressed in this article are solely those of the authors and do not necessarily represent those of their affiliated organizations, or those of the publisher, the editors and the reviewers. Any product that may be evaluated in this article, or claim that may be made by its manufacturer, is not guaranteed or endorsed by the publisher.

## Footnotes

^1^ https://www.ebi.ac.uk/Tools/psa/emboss_water/

^2^ https://services.healthtech.dtu.dk/service.php?SignalP-5.0
