# Supplementary material for: A Periplasmic Lanthanide Mediator, Lanmodulin, in Methylobacterium aquaticum Strain 22A
Source: Front Microbiol. 2022 Jun 23;13:921636. doi: 10.3389/fmicb.2022.921636 (PMC9260416; doi:10.3389/fmicb.2022.921636)

Supplementary file
Fujitani, Y., Shibata, T., and Tani, A.

A periplasmic lanthanide mediator, lanmodulin, in *Methylobacterium aquaticum* strain 22A

Yoshiko Fujitani, Takeshi Shibata, and Akio Tani

Fig. S1. Assessment of P_{fae1} and P_{mxaF} activity using a GFP-reporter. P_{fae1} and P_{mxaF} were each cloned into a GFP-reporter vector pHC42m, and the plasmids were introduced into strain 22A wild type. Their activity was assessed by measuring GFP fluorescence in strain 22A grown on methanol in the absence and presence of 30 µM La^{3+}. Strain 22A carrying pHC42m was regarded as control. The data are taken at 19 h. Data are presented as mean value ± standard deviation (SD), and analyzed with two-way analysis of variance (ANOVA) followed by a Tukey's multiple comparison test, and shown with compact letter display ($p < 0.05$, n = 3).

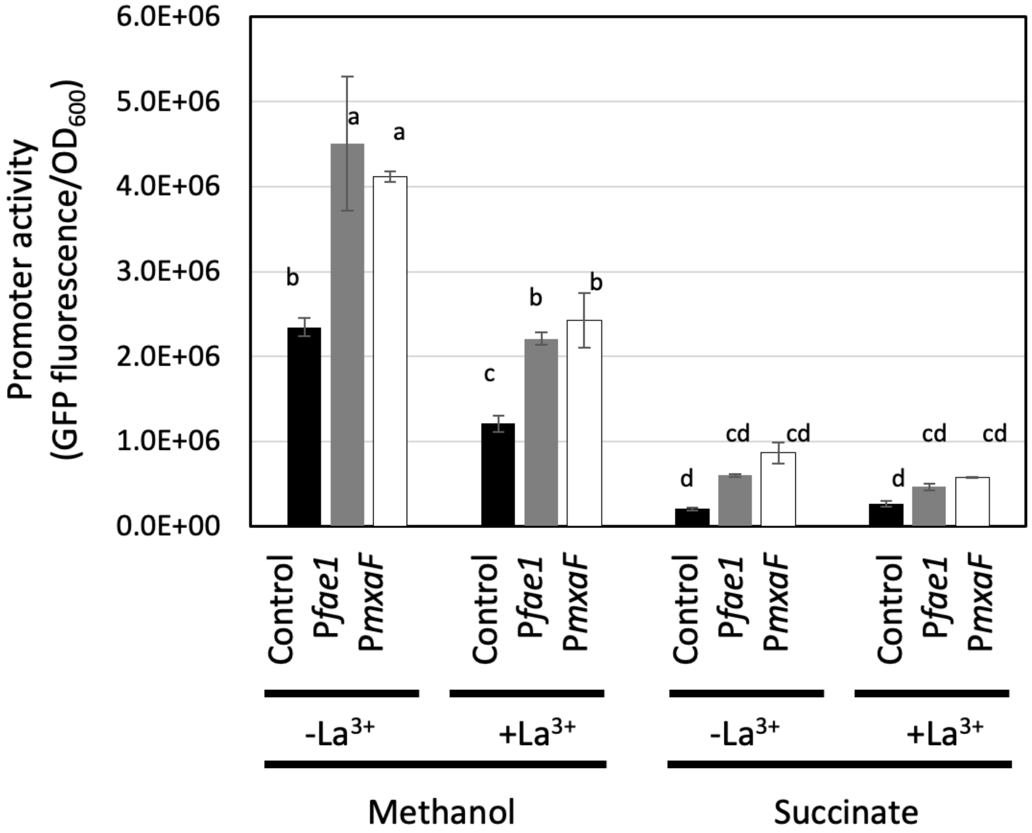

Supplementary file

Fujitani, Y., Shibata, T., and Tani, A.

Fig. S2. A. Gene organization of a chromosomal region containing *lanM* in the *M. aquaticum* strain 22A genome. Arrows indicate gene orientation and size. The suffix of the accession numbers (Maq22A_) is not shown. B. Alignment of LanM amino acid sequences from strains AM1 and 22A. The putative signal peptide sequences are shown in bold. The EF-hand motif (D(or E, N)xDxDxxxxxxE) is shaded.

A

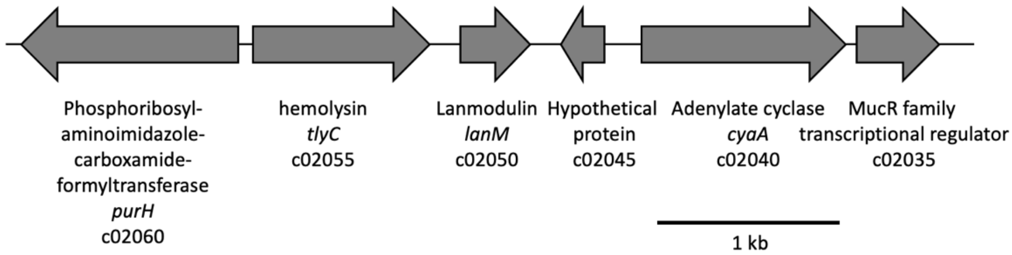

B

AM1 **MAFRLSSAVLLAALVAAP------AYAA**PTTTTKVDIAAFDPDKDGTIDLKEALAAGSAAFDKLDPDKDGTLDAKE 70

22A **MLNRTLLLAALLAAPLALPASA**EAKPAADR–TIAAVDTDSDGTLDLAEVKAAAAAVFDKLEKDADGTLDAKE 71

AM1 LKGRVSEADLKKLDPDNDGTLDKKEYLAAVEAQFKAANPDNDGTIDARELASPAGSALVNLIR 133

22A LKGRLSRQQLMKADPDNDGTLTKDEYVALAESLFKAADPDNDGTLDAKEMKSPAGKTLARLIK 134

Fig. S3. Heterologous expression of His-LanM and GST-LanM in *E. coli.* A. Expression of His-LanM in *E. coli* DH5α (pCold-*lanM*) and purification. Lane 1, Protein marker; 2, *E. coli* DH5α (pCold-*lanM*) cell-free insoluble fraction; 3, *E. coli* DH5α (pCold-*lanM*) cell-free extract; 4, Flow-through fraction of Ni-NTA column; 5-9, elution fractions (1 ml each). Samples were analyzed by SDS-PAGE (18.2% gel). Theoretical mass of His-LanM is 14.0 kDa. B. Expression of GST-LanM in *E. coli* DH5α (pGEX-*lanM*). 1, Protein marker; 2, *E. coli* DH5α (pGEX-*lanM*) cell-free extract. The sample was analyzed by SDS-PAGE (10% gel). Theoretical mass of GST-LanM is 36.7 kDa. C. Purification of LanM cleaved from GST-LanM. 1, Protein marker; 2, LanM protein eluted from GST-ACCEPT by PreScission protease. The sample was analyzed by SDS-PAGE (18.2% gel). Theoretical mass of LanM is 12.3 kDa.

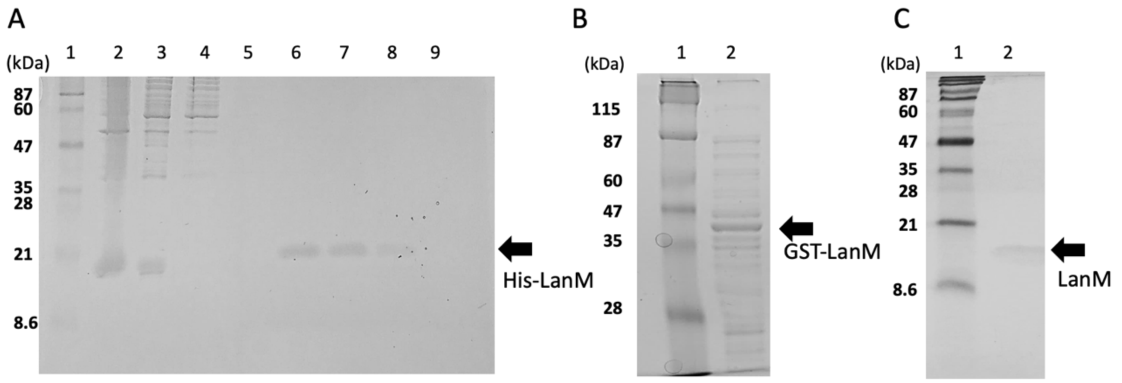

Fig. S4. Specific growth rate of various mutants on methanol and succinate in the absence/presence of $LaCl_3$. Data are presented as the mean value ± standard deviation (SD) (n = 3).

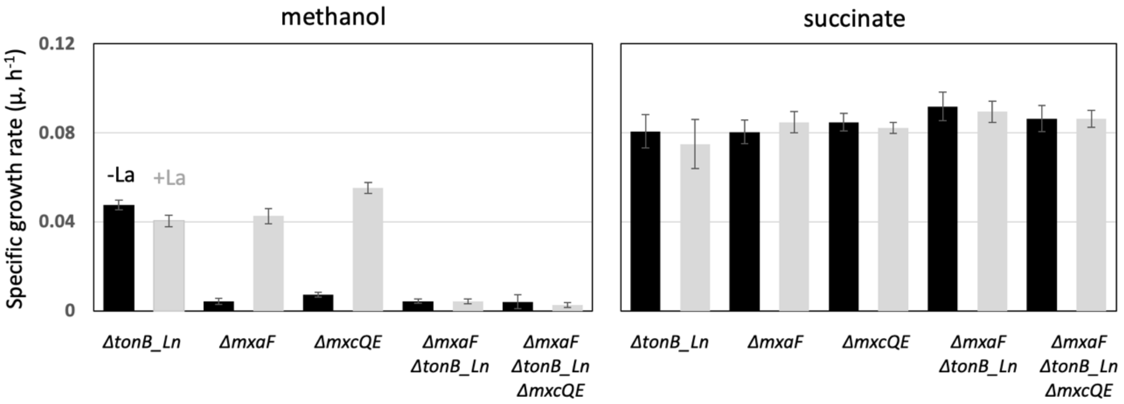

Fig. S5. SWATH proteome analysis of the wild-type strain 22A and Δ*lanM* grown on methanol in the absence/presence of $LaCl_3$. The peptide peak area of the identified and detected proteins are shown as a heatmap.

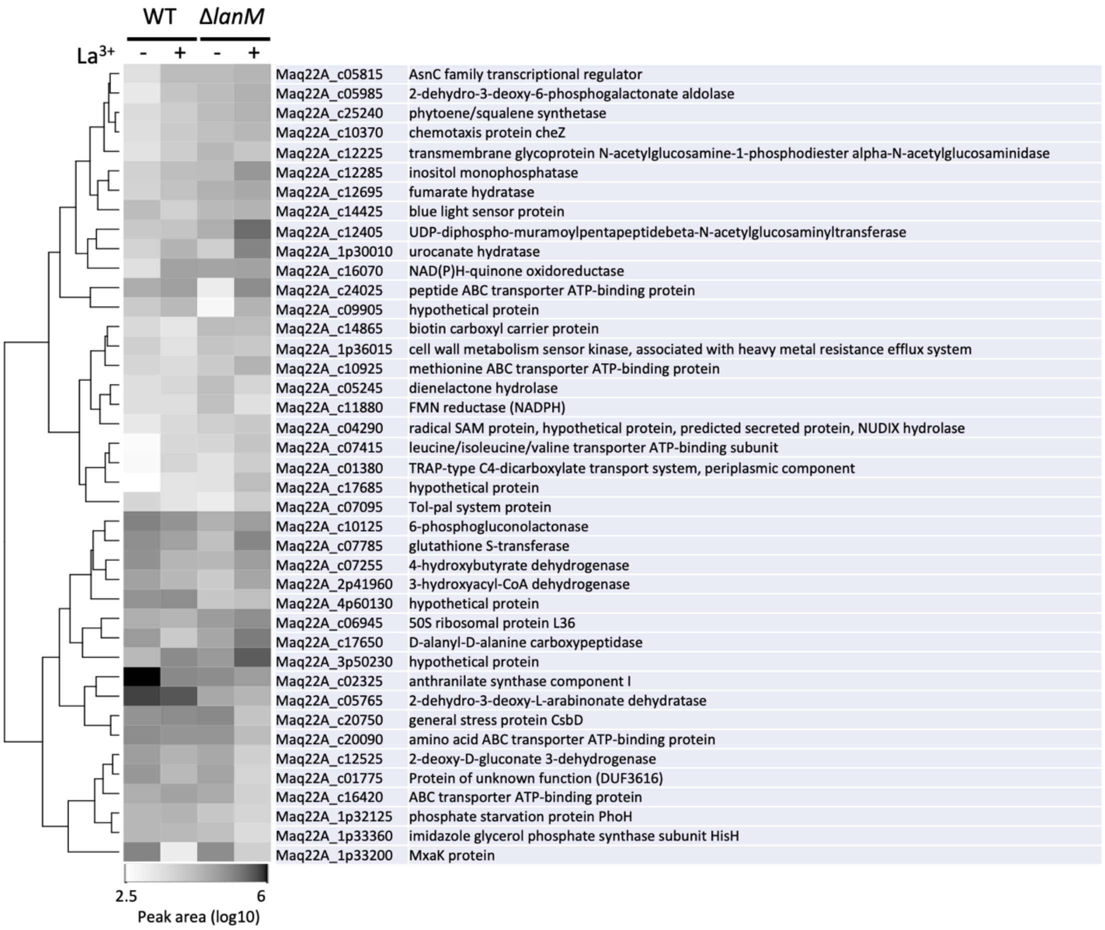

Fig. S6. Confocal microscopic images of MVs from *ΔlanM*, wild-type, and lanM-OX strains grown on methanol in the presence of 30 μM $LaCl_3$. MVs collected by a commercial kit (upper panel) and by ultracentrifugation (lower panel) are shown. Bar, 2 μm.

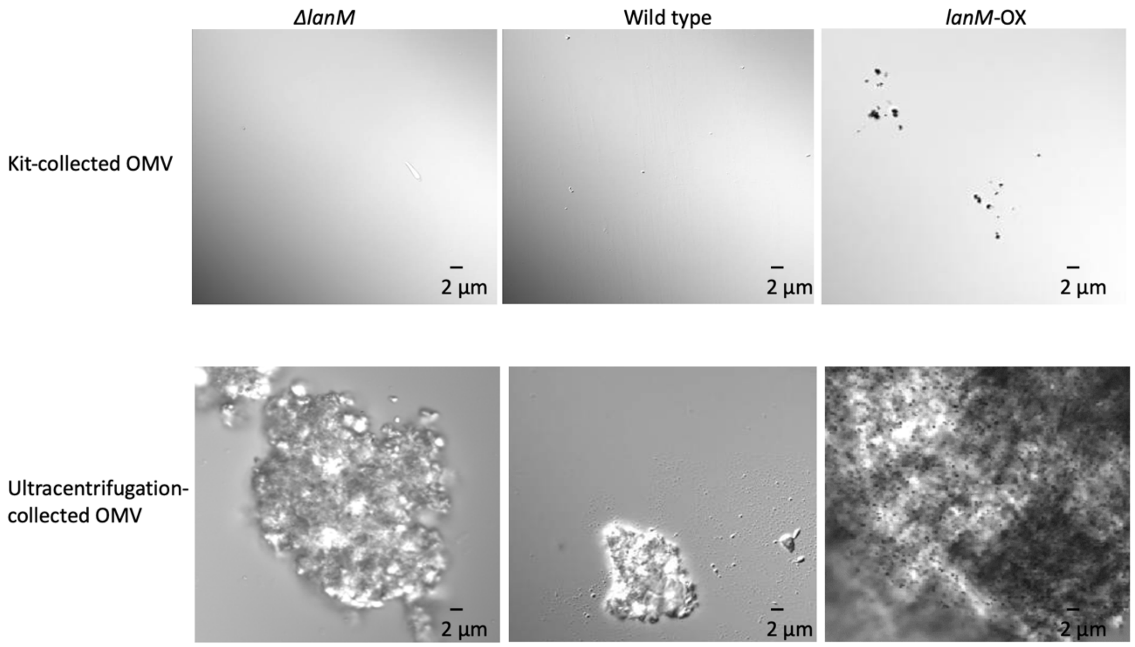

Fig. S7. La content in the MV fractions collected by a commercial kit from the wild-type, Δ*lanM,* and *lanM*-OX strains. The data were analyzed with one-way ANOVA followed by Tukey’s multiple comparisons test. Data are presented as the mean value ± standard deviation (SD) (n = 3).

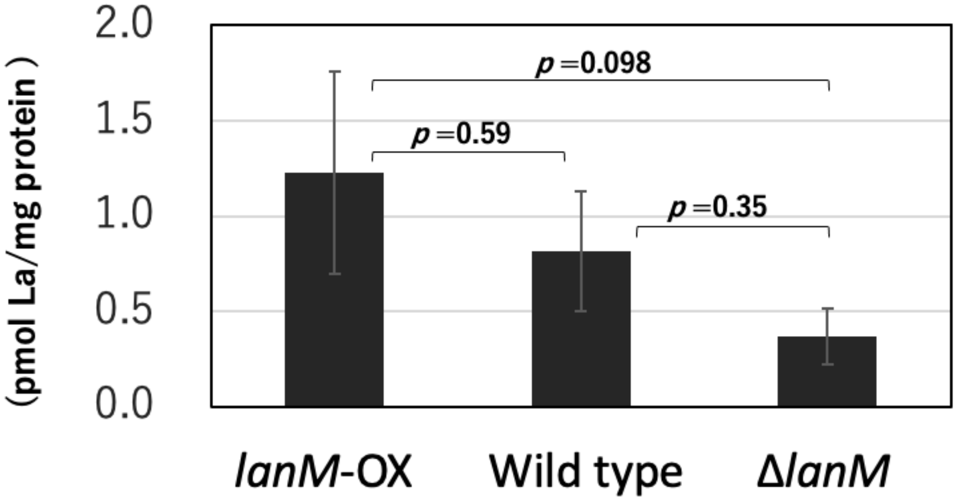

Supplement: Supplementary file 1 [file Data_Sheet_1.PDF]
